# Book Reviews

**Published:** 1911-12

**Authors:** 


### BOOK REVIEWS

MANUAL OF THE DISEASES OF THE EYE. For Students and General Practitioners. By Chas. H. May, M. D., Chief of Clinic and Instructor in Opthalmology, College of P. & S., Medical Dept. Columbia University, New York. Seventh Edition. Revised, with 362 original illustrations, 22 plates and 62 colored figures. Price $2.00. Wm. Wood & Co., N. Y.

This book should be taken as a type for books for medical students. It is just the right size, is well arranged and splendidly illustrated. Better physicians will graduate when more or all of the branches are studied from such books as this.

---

THE PRACTITIONER'S VISITING LIST FOR 1912. An invaluable pocket-sized book containing memoranda and data important for every physician, and ruled blanks for recording every detail of practice. The Weekly, Monthly and 30-Patient Perpetual contain 32 pages of data and 160 pages of classified blanks. The 60-Patient Perpetual consists of 256 pages of blanks alone. Each in one wallet-shape book, bound in flexible leather, with flap and pocket, pencil with rubber, and calendar for two years. Price by mail, postpaid, to any address, $1.25. Thumbletter index, 25 cents extra. Descriptive circular showing the several styles sent on request. Lea & Febiger, Publishers, Philadelphia and New York.

The text portion of The Practitioner's Visiting List for 1912 has been thoroughly revised and brought up to date. It contains, among other valuable information, a scheme of dentition; tables of weights and measures and comparative scales; instructions for examining the urine; diagnostic table of eruptive fevers; incompatibles; poisons and antidotes; directions for effecting artificial respiration; extensive table of doses; an alphabetical table of diseases and their remedies, and directions for ligation of arteries.

---

A TEXT-BOOK UPON THE PATHOGENIC BACTERIA. By Joseph McFarland, M. D., Professor of Pathology and Bacteriology in the Medico-Chirurgical College of Philadelphia. Octavo of 709 pages, illus. Cloth. $3.50 net.

In this edition the entire work has been thoroughly revised, old matter eliminated, much new matter inserted, and the subjects treated brought up to date. The value of the work has been considerably enhanced by the introduction of a large number of references to the literature.

---

MODERN SURGERY. General and Operative. By John Chalmers DaCosta, M. D., Professor of Surgery and of Clinical Surgery in the Jefferson Medical College, Philadelphia. Fifth Revised Edition; much enlarged and reset. Handsome octavo of 1283 pages, with 872 illustrations. Cloth, $5.50 net; sheep or Half Morocco, $7.00 net.

For this new fifth edition the work has been entirely rewritten and reset. One hundred new practical illustrations have been added, and the work has been enlarged by the addition of two hundred pages. Some of the subjects added are: Scopolamin-morphin anaesthesia; local anaesthesia by injection of stovain; Willy Meyer's operation for carcinoma of the mammary gland; Young's perineal prostatectomy; the Johns Hopkins operation for inguinal hernia; the Quene-Mayo operation for rectal cancer; Moynihan's short loop method of gastro-jejunostomy; and appendicostomy.

POCKET THERAPEUTICS AND DOSE-BOOK. With collateral information of much value to the practitioner. By Morse Stewart, Jr., M. D. 32mo of 263 pages.

This book is a complete therapeutics as well as a dose-book, and it is so arranged that the information sought can be obtained at a glance. Its contents include a classification and explanation of the actions of medicines, dosage (both Troy and metric sys tems), index of diseases with appropriate remedies, tables of solubilities, prescription-writing, poisions, their symptoms, antidotes, and treatment, incompatibles and antagonists and much other information.

---

A TREATISE IN DIAGNOSTIC METHODS OF EXAMINATION. By Prof. Dr. H. Sahli, of Bern. Edited, with additions, by Francis P. Kinnicutt, M. D., Professor of Clinical Medicine, olumbia Universty, N. Y.; and Nathaniel Bowditch Potter, M. D., Associate in Clinical Medicine at Columbia University, N. Y.; and Consulting Physician to the Manhattan State Hospital, N. Y. Octavo of 1008 pages, profusely illustrated. Cloth, $6.50 net; Half Morocco, $8.00 net.

Sahli's splendid work upon this work has been recognized as the best since its publication in German and cannot be too highly praised for its excellence.

---

DISEASES OF CHILDREN FOR NURSES. By Robert S. McCombs, M. D., Instructor of Nurses at the Children's Hospital of Philadelphia. 12mo of 431 pages, illustrated, including a number of colored plates. Cloth, $2.00 net.

This book gives much valuable information to the nurse, who often has more to do with the success or failure of treatment of children than the physician, for upon her devolves the important matter of carrying out instructions.

---

THE FOUR EPOCHS OF WOMAN'S LIFE: A Study in Hygiene, Maidenhood, Marriage, Maternity, Menopause. By Anna M. Galbraith, M. D. With an introductory Note by John H. Musser, M. D., University of Pennsylvania. 12mo of 247 pages. Cloth $1.50 net.

Galbraith gives much wise and wholesome advise to women and the book can go with safety to intelligent female patients.

